# Dietary Bioactive Compounds and Human Health: The Role of Bioavailability

**DOI:** 10.3390/nu17010048

**Published:** 2024-12-27

**Authors:** Italo Rennan Sousa Vieira, Carlos Adam Conte-Junior

**Affiliations:** 1 Analytical and Molecular Laboratorial Center (CLAn), Institute of Chemistry (IQ), Federal University of Rio de Janeiro (UFRJ), Cidade Universitária, Rio de Janeiro 21941-909, RJ, Brazil; 2 Center for Food Analysis (NAL), Technological Development Support Laboratory (LADETEC), Federal University of Rio de Janeiro (UFRJ), Cidade Universitária, Rio de Janeiro 21941-598, RJ, Brazil; 3 Laboratory of Advanced Analysis in Biochemistry and Molecular Biology (LAABBM), Department of Biochemistry, Federal University of Rio de Janeiro (UFRJ), Cidade Universitária, Rio de Janeiro 21941-909, RJ, Brazil

The relationship between dietary bioactive compounds and human health has generated significant interest among members of the scientific community and the general public. These dietary phytochemicals encompass a diverse group, including polyphenols, carotenoids, phytosterols, omega-3 fatty acids, probiotics, prebiotics, glucosinolates, lignans, and flavonoids, which offer potential health benefits such as antioxidant and anti-inflammatory properties and the potential for preventing or treating chronic diseases [1,2,3]. However, several factors influence their solubility and bioavailability, such as their molecular structure, food matrix effects, transporters, pH variations, and gut microbiota metabolism [4,5]. The bioavailability of these compounds is critical to understanding their potential health benefits [6]. Recent strategies have been employed to overcome this challenge, such as structural modifications, colloidal systems, and nanotechnology, aiming to increase the absorption and bioavailability of bioactive compounds [7,8,9]. Furthermore, emerging research is paving the way for personalized dietary recommendations, considering individual metabolic and genetic factors, thereby promoting more targeted approaches to nutrition and health [10,11]. The following Special Issue (SI), entitled “Dietary Bioactive Compounds and Human Health: The Role of Bioavailability”, brings together high-quality studies that explore the mechanisms of bioavailability and innovative approaches to enhance the benefits of bioactive compounds to human health.

This SI includes six publications, comprising two original research articles and four literature reviews, addressing various aspects related to the absorption and metabolism of bioactive compounds in the human body, in addition to applications of nanotechnology and nanotherapeutics to improve the bioavailability, clinical efficacy, and delivery systems of bioactive compounds.

Of the included articles, the authors of both in vitro and in vivo studies evaluate the effect of plant-derived bioactive compounds on digestion and chronic disease therapy:

Nekvapil et al. (2024) (Contribution 1) investigated the release of organic acids and low-molecular-weight carbohydrates from *Camellia sinensis* (Matcha) during in vitro digestion. Approximately 67.3% and 85.9% of the dry matter was digested after simulated gastric and intestinal digestion, respectively. The primary organic acids identified were citric, malic, oxalic, and succinic acids, with malic acid being ultimately released and succinic acid being partially retained. Among the carbohydrates, trehalose predominated (36.1 mg/g), with 87% released, leaving the remainder to reach the large intestine. Despite potential benefits, such as modulating the gut microbiota and nutrient bioavailability, the high oxalic acid content, intensified by shading techniques, may pose risks for individuals susceptible to kidney stones, necessitating further studies to establish safe consumption limits.

In a clinical investigation, Venugopal et al. (2024) (Contribution 2) examined the metabolic effects of GLUBLOC^TM^, a nutraceutical supplement based on *Morus alba* (white mulberry) and *Malus domestica* (apple) extracts, on postprandial glucose and insulin levels in healthy individuals. The results of their randomized, crossover clinical trial demonstrated that GLUBLOC^TM^ supplementation before a carbohydrate-rich meal or sucrose beverage significantly reduced glucose and insulin spikes. The results suggested that GLUBLOC^TM^ may limit glucose absorption by inhibiting digestive enzymes such as α-amylase and α-glucosidase, offering potential as an adjunct in managing postprandial glucose, particularly in high-carbohydrate diets.

From reviewed studies, Pi et al. (2024) (Contribution 3) examined the relationship between bioactive polysaccharides (BPs) from natural sources and the gut microbiota in metabolic diseases, such as obesity, type II diabetes mellitus, non-alcoholic fatty liver disease, and cardiovascular diseases. BPs derived from plants, seaweed, and fungi exhibit antioxidant, immunomodulatory, and metabolic regulatory activities, with them being metabolized by the gut microbiota into beneficial compounds such as short-chain fatty acids. The authors emphasize that alterations in microbiota composition are linked to metabolic disease progression and that BPs may restore microbial balance, reduce inflammation, and improve metabolic indicators such as glucose and lipid levels. While promising as natural therapies, BPs require further examination to understand their clinical efficacy and safety.

The authors of the other included studies reviewed aspects of bioavailability and nanotechnology strategies to increase the therapeutic efficacy of bioactive compounds for maintaining human health:

Speers et al. (2024) (Contribution 4) provided a comprehensive review of pharmacokinetic studies and analytical methods for quantifying withanolides in plasma following *Withania somnifera* (ashwagandha) administration. Withanolides such as withaferin A and withanolide A were analyzed in animal models and humans, primarily using liquid chromatography–mass spectrometry (LC-MS). Plasma concentrations varied widely depending on the compound, dose, and administration route. The results of clinical studies indicated low systemic levels of withanolides, highlighting the need for further research to associate preclinical data with clinical applications. Furthermore, the study authors highlight some analytical limitations, such as variability in detection limits, emphasizing the need for standardization in future research.

In another study, the effect of polyphenolic compounds, such as resveratrol, epigallocatechin, and curcumin, in the treatment of osteoarthritis was investigated by Hridayanka et al. (2024) (Contribution 5). The study authors highlight the main limitations of conventional therapies, such as low bioavailability and adverse effects, and suggest nanotherapeutic strategies to improve the targeted delivery, efficacy, and controlled release of the compounds. Furthermore, the authors discuss the roles of epigenetics, including histone modifications and the expression of noncoding RNAs, in controlling inflammation and protecting cartilage. The interaction between the gut microbiota and epigenetics was also examined, emphasizing its influence on the development and progression of osteoarthritis.

Lastly, Altemimi et al. (2024) (Contribution 6) explored the potential of nanotechnology to enhance the bioavailability, absorption, and stability of bioactive compounds in human nutrition. The authors demonstrated how various systems, including nanoemulsions, nanofibers, and nanocomposites, can encapsulate vitamins, minerals, and antioxidants, enabling controlled nutrient and bioactive compound delivery and contributing to more effective functional foods. Despite these advantages, the authors raise concerns about the safety and toxicity of nanoparticles, emphasizing the need for regulatory standardization and broader studies on their impact on human health and the environment.

The studies included in this Special Issue address the relationship between bioactive compounds, their bioavailability, and their potential effects on human health. Studies focused on digestion, metabolism, and application of nanotechnology approaches reinforce the need for integrated research to increase the therapeutic potential of dietary bioactive compounds. Future research should focus on increasing bioavailability through nanotechnology and personalized nutrition strategies [12], supported by advancements in omics technologies to develop tailored nutritional recommendations based on individual genetic and metabolic profiles [13,14]. Further investigation into the long-term effects of nanocarriers of bioactive compounds and nutrients is crucial to ensure safety and efficacy for clinical applications and functional foods.
